# Transcriptomic analysis of heteromorphic stamens in *Cassia biscapsularis* L.

**DOI:** 10.1038/srep31600

**Published:** 2016-08-16

**Authors:** Zhonglai Luo, Jin Hu, Zhongtao Zhao, Dianxiang Zhang

**Affiliations:** 1 Key Laboratory of Plant Resources Conservation and Sustainable Utilization, South China Botanical Garden, the Chinese Academy of Sciences, Guangzhou 510650, China; 2 Shenzhen Park Service, Shenzhen 51800, China

## Abstract

Hermaphroditic flowers have evolved primarily under the selection on male function. Evolutionary modification often leads to stamen differentiation within flowers, or “heteranthery”, a phenomenon intrigued scientists since the 18^th^ century until recently. However, the genetic basis and molecular regulation mechanism has barely been touched. Here we conducted comparative transcriptome profiling in *Cassia biscapsularis* L., a heterantherous species with representative patterns of stamen differentiation. Numerous differentially expressed genes (DEGs) were detected between the staminodes (the degenerated stamens) and fertile stamens, while much fewer genes differentially expressed among the three sets of fertile stamens. GO term enrichment and KEGG pathway analysis characterized functional properties of DEGs in different stamen types. Transcripts showing close correlation between expression pattern and stamen types were identified. Transcription factors from the bHLH family were suggested to have taken crucial part in the formation of staminodes. This first global transcriptomic analysis focusing on stamen differentiation opens the door toward a more comprehensive understanding on the molecular regulation of floral organ evolution. Especially, the generated unigene resource would be valuable for developing male sterile lines in agronomy.

Flower form evolution played key role in angiosperm radiation^1^,^2^. The “enigmatic” diversity of angiosperm floral organs has intrigued both the botanists and evolutionary biologists ever since the 18^th^ century^3^. Stamens are the male reproductive organs of flowering plants with the primary functions of pollen production and presentation. From an evolutionary perspective, hermaphroditic flowers have evolved primarily as pollen-donating rather than pollen-receiving organs^4^,^5^. In other words, the evolution of floral traits was driven primarily by selection on male function. During the evolution of floral forms, androecia (the set of a flower’s stamens) modification frequently resulted in stamen repression, stamen loss or transformation of stamen function^6^. This leads to intrafloral stamen differentiation (differentiation of stamens within flowers), and the formation of heteromorphic stamens, or “heteranthery”, a within-flower polymorphism documented in many unrelated families^7^,^8^.

Heteranthery, the presence of stamens in a flower that differ notably in size, colors and/or shape, and presumably with different functions, was first described more than one hundred years ago^9^. Darwin hypothesized that it reflects a “division of labor” among stamens^9^. One set of stamens would satisfy the pollinators’ demand for pollen as food (the “feeding” stamens), the other meet the plant’s need for safe gamete transport (“fertilization” stamens). Stamen dimorphism, one of Darwin’s last scientific enquiries, intrigued him for more than 20 years^10^. Due to the lack of direct experimental evidence for the respective roles of each stamen set, debate on the “division of labor” hypothesis has lasted for over one century. The puzzle has been solved recently^11^,^12^. Taking advantage of the natural morphological difference between the pollen grains from the two sets of stamens, Luo *et al*.^11^ tested Darwin’s hypothesis in the heterantherous *Melastoma malabathricum* (Melastomataceae) and a division of labor amongst the stamen types has been substantiated in these flowers. The study provided the first unequivocal support for the “division-of-labor” hypothesis explaining stamen heteromorphism^11^. Similar to the dimorphic stamens in *Melastoma malabathricum*, androecia can differentiate into three (e.g. *Cassia didymobotrya, Swartzia trimorphica*), four (e.g. *Cassia biscapsularis, C. siamea*) or even more sets of stamens in some taxa^7^,^13^,^14^. On a broader scale, Luo and co-authors^7^ analyzed and compared stamen differentiation in seven heterantherous species. Differed differentiation patterns were reported, while pollen grain number was found to be positively related to anther sizes. However, all the studies so far have focused on the morphological and functional aspects, while barely touched the genetic basis of heteranthery.

The modification of androecia sometimes results in sterile stamens, or staminodes, which have lost the function of producing fertile pollen^6^. In the heterantherous Commelineae (Commelinaceae), the evolution of fertile stamens to sterile “feeding” stamens has occurred as part of a pollen mimicry system, that the staminodes function as visual attractant for pollinators^15^. Sterile stamens may also take the role of providing rewards, avoiding self-pollination, while in many taxa, the function of staminodes is still unknown^6^.

The origin of angiosperm flowers has been referred to as an ‘abominable mystery’ by Darwin^16^. Flower development has served as one important model system over the past three decades in the study of the molecular control of organogenesis in angiosperms^17^. Genetic and molecular analysis in model plants have identified a large number of floral regulators involved in the control of flower development^18^,^19^,^20^. Thousands of anther-specific transcripts have been identified in *Arabidopsis*, however, the functions or expressions of most of these transcripts are still unclear^21^,^22^,^23^. The molecular mechanisms behind intrafloral stamen differentiation, especially those for the morphological and functional differentiations of fertile stamens in non-model plants, await to be explored.

Next-generation sequencing (NGS) provides a novel approach for exploring transcriptomic data with distinct advantages in resolution, robustness and inter-lab portability, compared with previous techniques like the microarray platforms^24^. NGS technology is especially helpful for comparative analysis of RNA-Seq data in non-model species with limited genomic information^25^. Comparative transcriptome analysis has been successfully used to explore candidate genes in economic species, such as cotton and eggplant^26^,^27^. Nowadays, RNA-Seq built on NGS technology has become the most powerful tool for transcriptomic data mining.

From the perspective of agricultural economy, the molecular mechanisms underlining stamen sterility are of special significance for agronomic questions related to male sterility. In pummelo, transcripts encoding key transcription factors for stamen identification were found to be restricted to normal floral whorls, while repressed in the sterile line^28^. And important DEGs and biological processes were reported to contribute to the sterile trait in cotton^29^.

*Cassia sensu lato,* with the greatest androecial diversity among Cassiinae (Fabaceae)^30^,^31^, is prominent for its unique androecial patterns and poricidal anthers (anthers that dehisce through terminal pores), which have fascinated botanists for a long time^32^,^33^. The form and structure of *Cassia* stamens has been studied by Marazzi *et al*.^30^ and Marazzi & Endress^34^. Luo *et al*.^7^ have investigated the intrafloral differentiation of stamens in *C. biscapsularis*, paying special attention to the morphological difference of androecia, palynological characteristics, as well as pollinators’ behavior. These studies have provided basic information for our present research. Here we conducted comparative transcriptomic study of different stamen sets in the heterantherous *Cassia biscapsularis* L. As the androecia comprise of both staminodes and fertile stamens with notable morphological differences, it offers an idea model to explore the molecular mechanisms behind intrafloral stamen differentiation. Through this research, we aim to: (1) developing unigene datasets and exploring possible transcriptional specialization for different sets of stamens; (2) screening candidate genes and biological pathways associated to stamen differentiation during organ maturation. Our study provides the first comprehensive global-scale transcriptomic profiling on the formation of heteranthery. The data will place the foundation for further functional analysis of stamen development, and the molecular resources will be valuable for elucidating the molecular factors governing male sterility in both botanical and agronomic researches.

## Results

### Anatomical characteristics of staminodes vs. fertile stamens in *Cassia bicapsularis*

**Figure 1:**
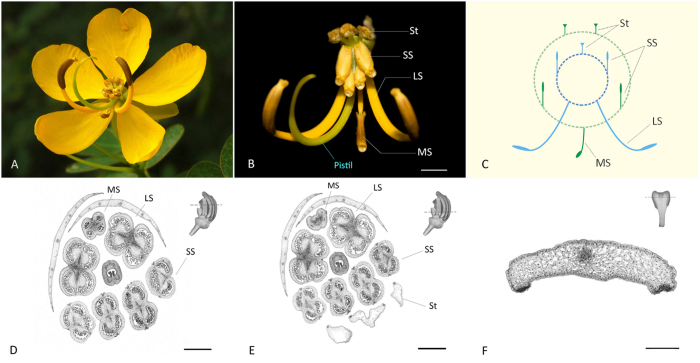
(**A**) Fully opened flower of *Cassia biscapsularis* L. in natural condition; (**B**) Flower with petals removed showing the four sets of stamens (LS: long stamens, MS: middle stamen, SS: short stamens, St: staminodes) and the pistil. (**C**) Schematic diagram illustrating the arrangement of the two whorls of stamens. (**D**) Transverse section of small flower bud showing the developing normal microspores in the anthers of fertile stamens (LS, MS and SS); (**E**) Transverse section at a lower level, and no microspores present in the staminodes (St); (**F**) Transverse section of staminode in mature flower bud showing the absence of pollen in anther sac. Three petals were removed in (**D,E**). Scale bar = 5 mm in (**B**) 500 μm in (**D,E**) 100 μm in (**F**).

The androecia of *C. bicapsularis* have four types of stamens with notable morphological differentiation (Fig. 1A–C). The three adaxial stamens (staminodes, St for short) are sterile and flattened. At the center of the flower there are four short stamens (SS for short) with erect filaments and yellow anthers, which were suggested as “feeding” stamens with the function of providing pollen as food for larvae of pollinators^7^,^13^. The three abaxial stamens (“pollinating” stamens) form two sets. The two lateral abaxial stamens (long stamen, LS for short) have long, curved filaments and brown anthers, while the third stamen (middle stamen, MS for short) is centric and lowermost, with shorter filament and brown anther.

The three fertile stamen sets showed normal and similar microsporogenesis and male gametophyte development. In flower buds of approx. 5 mm in diameter, the locules of fertile stamens were filled with developing microspores, while tapetum or microspores were completely absent in the staminodes (representative images were shown in Fig. 1D,E). And in mature staminodes, parenchyma cells loosely filled the anther sac (Fig. 1F).

### Sequence analysis and *de novo* assembly

On average, approximately 94 million raw pair-end reads were generated for each library by Illumina HiSeq™ 2000 sequencing. After data filtering and stringent quality check, over 80 million clean reads with >97% Q20 bases were selected per library as high quality reads for further analysis.

The short clean reads were assembled into unigenes by Trinity^35^ (78,773 for Cb-L, 84,297 for Cb-M, 80,063 for Cb-S, 78,120 for Cb-St), with a mean length of 718 bp, 674 bp, 658 bp, and 704 bp respectively (Table 1). A total of 80,380 non-redundant unigenes (All-Unigenes) were produced, with a mean length of 979 bp and N50 of 1564 bp. Among these unigenes, 53,404 (66.44%) unigenes were longer than 500 bp, and 31,970 (39.77%) were longer than 1000 bp (Supplementary Fig. S1).

### Functional annotation of unigenes

**Figure 2:**
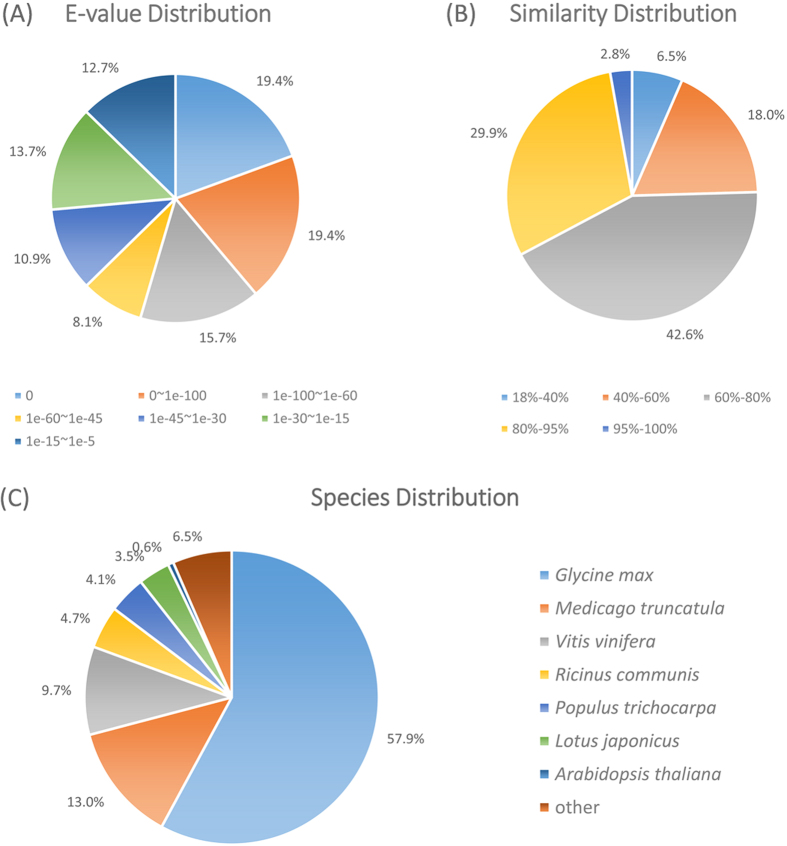
Characteristics of sequence homology search against the NR database. (**A**) E-value distribution of BLAST hits for matched unigene sequences, using an e-value cutoff of 1.0E-5; (**B**) Identity distribution of top BLAST hits for each unigene. (**C**) Species distribution of the top hits in the NR database.

Functional annotation, consists of protein functional annotation, pathway annotation, COG functional annotation and Gene Ontology (GO) annotation, provides important information on gene function. Unigene sequences were firstly aligned by blastx to protein databases NR, Swiss-Prot, KEGG and COG (e-value < 1.0E-5), and aligned by blastn to nucleotide databases NT (e-value < 1.0E-5). Approximate 72.9% (58,621) of the unigenes were annotated against different databases. The result of unigene annotation is summarized in Supplementary Fig. S2. For *Cassia biscapsularis*, the top hit species was *Glycine max*, followed by *Medicago truncatula* and then *Vitis vinifera* (Fig. 2), suggesting it’s more closely related to soybean.

**Figure 3:**
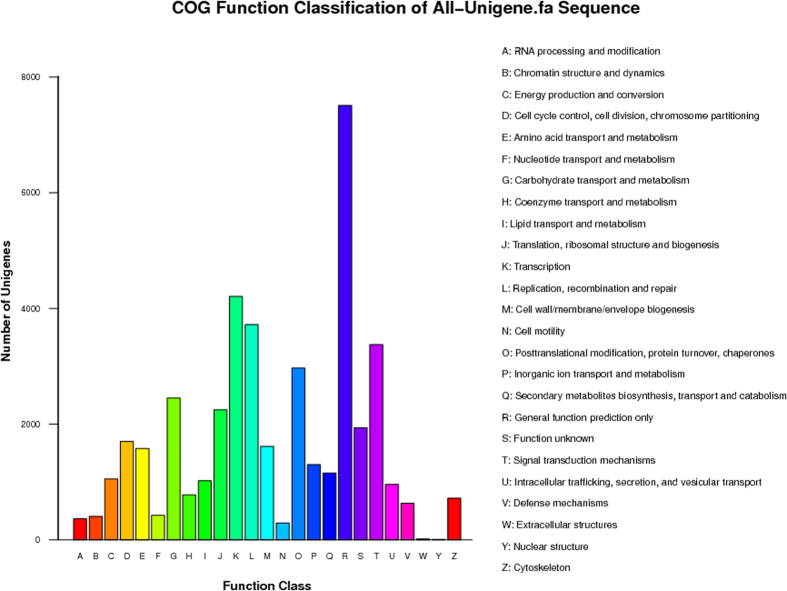
COG functional classification of unigenes.

Annotated unigenes were categorized into 25 functional groups according to the COG classification (Fig. 3). Among these COG categories, one-third (33.6%) of the unigenes were assinged to the clusters “transcription”, “replication, recombination and repair”, “signal transduction mechanisms” and “post-translational modification”, consistent with stamen’s function of producing male gametes.

### Gene ontology (GO) and metabolic pathway analysis

Based on the NR annotation, Gene Ontology (GO) analysis was performed. It provides a dynamic, controlled vocabulary and a strictly defined concept to comprehensively describe properties of genes and their products on molecular functions, cellular components and biological processes. Among the 56,403 unigenes annotated in NR, one or more GO terms were assigned to the 45,678 unigenes (Supplementary Fig. S3). For biological processes, the genes involved in “cellular process” (GO: 0009987) and “metabolic process” (GO: 0008152) were highly represented. The most represented category for cellular components was “cells” (GO: 0005623) and “cell parts”. For molecular functions, “binding activity” (GO: 0005488) was the most represented GO term, followed by the “catalytic activity” (GO: 0003824).

A total of 128 pathways were predicted from the KEGG database. Transcripts identified as related to the following cellular processes or components were the most abundant: metabolic pathways (6945 unigenes), biosynthesis of secondary metabolites (3386 unigenes), plant-pathogen interaction (2018 unigenes), and plant hormone signal transduction (1931 unigenes) (Supplementary Table S1).

### Analysis of differentially expressed genes (DEGs)

**Figure 4:**
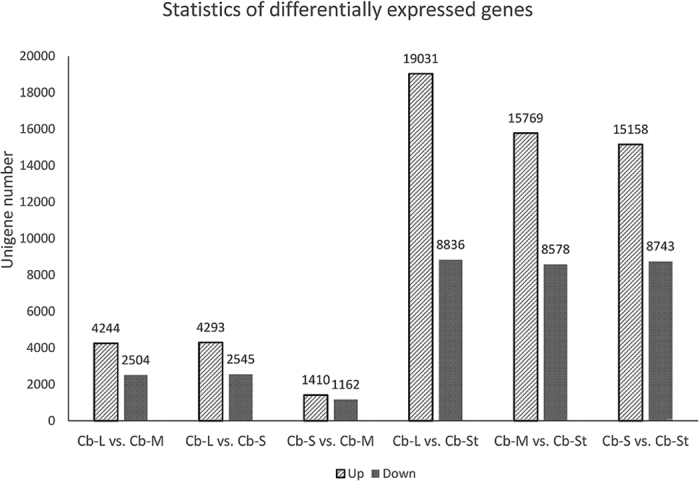
Differentially expressed genes between different stamen sets.

**Figure 5:**
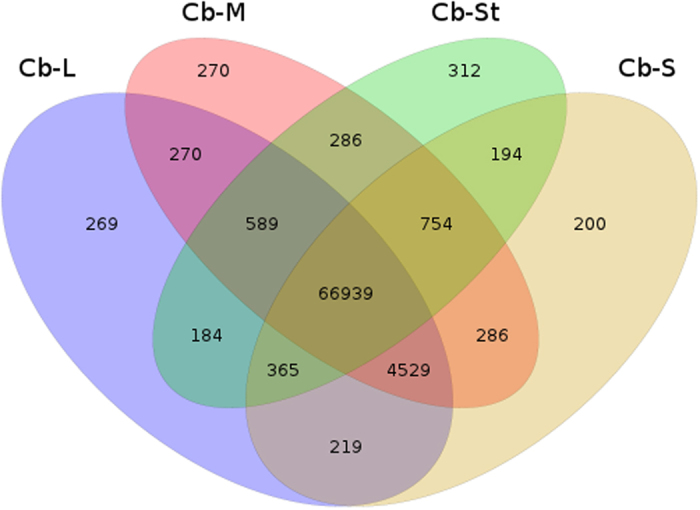
Venn diagram showing the number of transcripts expressed in the four sets of stamens. Some transcripts specifically expressed in certain type of stamens.

DEGs between different stamen sets were screened with thresholds fold change >2 and FDR ≤ 0.001. Tens of thousands of unigenes were found to be differentially expressed between the staminodes (St) and the fertile stamens (L, M, S). Compared with the staminodes, 19,031 unigenes were up-regulated in the long stamens; while 8,836 unigenes were down-regulated (Fig. 4). Similar patterns were also found between staminodes and the middle and short stamens. On the contrary, much fewer DEGs were detected among the fertile stamens, and the minimum difference was that between the middle and short stamens. Figure 5 further illustrates the number of expressed transcripts shared by different stamen sets, and some transcripts were stamen type-specific. The transcripts RPKM values of four stamen sets were accessed by box plotting, and many more transcripts in staminodes showed very low expression (RPKM ≤ 0.5) (Supplementary Fig. S4).

**Figure 6:**
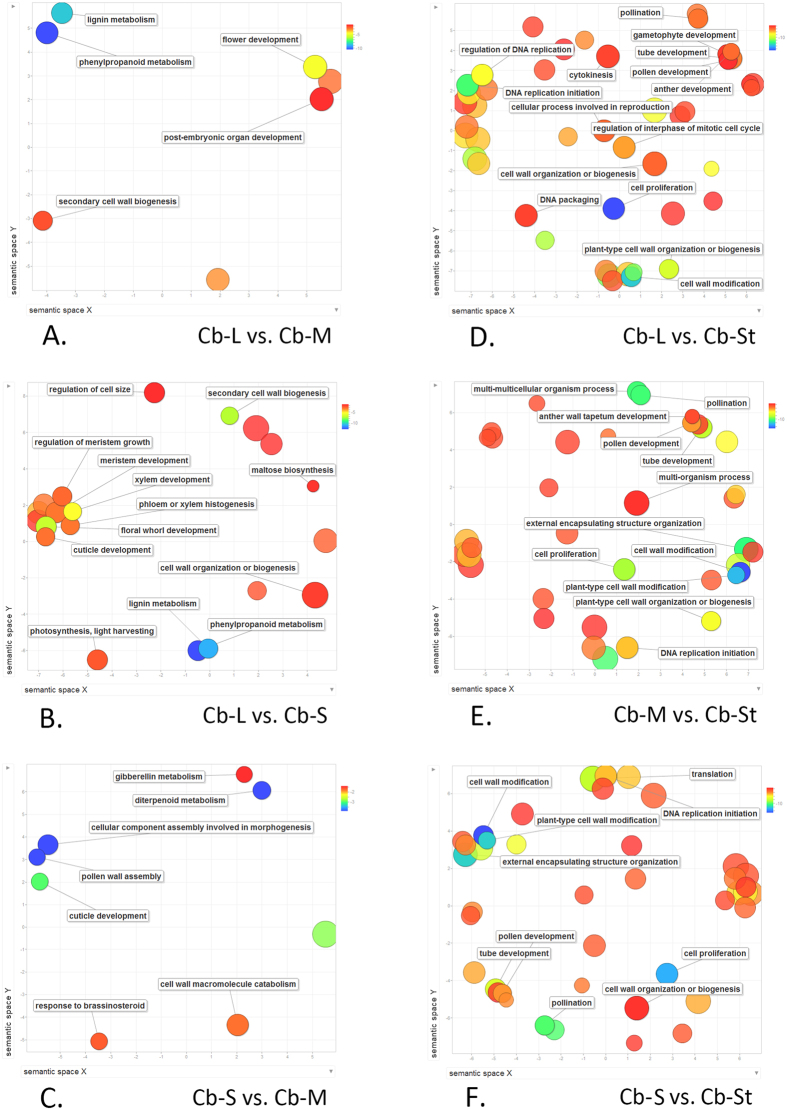
GO enrichment of DEGs between different stamen types. Scatterplot showing enriched GO terms between (**A**) long stamens and middle stamen; (**B**) long stamens and short stamens; (**C**) short stamens and middle stamen; (**D**) long stamens and staminodes; (**E**) middle stamen and staminodes; (**F**) short stamens and staminodes. The circle color represents the log10 transformed *p*-value in REVIGO analysis.

The most significantly enriched GO term in the fertile stamens compared with staminodes was “ribosomal subunit”, while “extracellular region” was the most represented GO term in the comparisons between fertile stamens (Supplementary Table S2). The biological processes of enriched GO terms in each comparison were visualized by ReviGO (Fig. 6). The amount and composition of GO terms differed significantly between comparisons. Many more enriched GO terms were identified in the comparisons between fertile stamens and the staminodes, compared with that between fertile stamen sets.

### Identification of stamen differentiation-related genes

**Figure 7:**
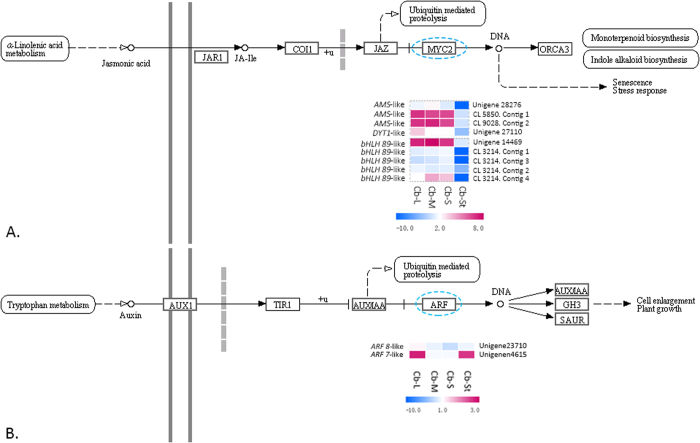
Plant hormone signal transduction pathway in *Cassia biscapsularis*. (**A**) Jasmonate signaling. Heat map shows the expression pattern of key transcripts encoding MYC2 in different types of stamens (log2-transformed RPKM). (**B**) Auxin signaling. Heat map shows the expression pattern of key transcripts encoding ARF (Auxin Response Factors) in different types of stamens (log2-transformed RPKM).

To investigate the genetic basis of intra-floral stamen differentiation, related DEGs were screened and analyzed coupling with the pathways they involved. We firstly focused on the transcriptomic changes between fertile stamens (L, M, S) and the staminodes (St), which exhibited the greatest difference. The expression patterns of certain transcription factor (TF) families differed significantly among stamen types (Supplementary Table S3). Most of the basic loop-helix-loop (bHLH) and bHLH-like TFs, a TF family that regulate tapetum-preferential genes, showed significantly higher expressions in the fertile stamens. They were significantly enriched (FDR ≤ 0.05) in all the three comparisons between fertile stamens (L, M, S) and the staminodes (St) (Supplementary Table S4). The bHLH family member ABORTED MICROSPORE (AMS)-like and *DYSFUNCTIONAL TAPETUM1 (DYT1*)-like TFs were highlighted for their key roles in regulating tapetum and microspore development^36^,^37^ (Supplementary Table S3). Pathway enrichment analysis further demonstrated that most of the screened *bHLH*-like genes were involved in the plant hormone signal transduction (ko04075), encoding MYC2 (K13422), which plays crucial role in the jasmonate (JA) signaling pathway (Fig. 7A).

Comparative analysis among the fertile stamen sets highlighted transcripts regulating cell growth and division. All three annotated auxin response factor (ARF)-like genes were significantly down-regulated in the M and S stamens, compared with the L stamens (Fig. 7B). Most plant growth and developmental processes are mediated through these ARF-involved gene expression^38^. A gibberellin 20 oxidase 2 (GA 20ox 2)-like gene (Unigene8068) was also identified for its significantly reduced expression from L to S stamens (Supplementary Fig. S5).

Most transcripts encoding MADS-box TFs and MADS-box proteins were significantly down-regulated in the staminodes (Supplementary Table S3). Two MADS-box transcription factor TM6 transcripts (CL9232.Contig1 and CL9232.Contig2), however, were significantly up-regulated in the staminodes. One ECE class CYC1 transcript (Unigene23017) was annotated, with highest expression in the staminodes and significantly reduced expression in fertile stamens (Supplementary Table S3).

## Discussion

Stamen development plays key role in angiosperm flower evolution, while the molecular regulations of which, however, remains under explored compared with other floral organs such as petals. Here we presented a comprehensive transcriptome profiling in the heterantherous *Cassia bicapsularis* to characterize gene expression in different stamen sets. The molecular signature in heteromorphic stamens would further our understanding of floral organ development.

### NGS-based RNA-Sequencing provided genome-wide expression profiles in heteromorphic stamens

The origin of heteranthery has been an “enigmatic” problem puzzling scientists for over one century. Taking advantage of the RNA-Seq technology, high-quality transcriptomic data were generated from the four stamen sets in *C. biscapsularis*. Over 80,000 non-redundant unigenes were produced, 58,621 of which could be identified as putative homologs of annotated sequences in public databases. The data represent the largest tissue-specific genetic resource for *Cassia* to date, a non-model plant with interesting biological characters. The results would greatly facilitate studies in the molecular regulation of flower development, especially those focusing on male organs.

### Comparative analysis revealed significant transcriptomic difference among stamen types

The diverse androecial differentiation patterns in heterantherous plants is one of the best examples illustrating floral adaptation to specialized pollination mode which has fascinated researchers for a long time^7^,^30^. The morphological specialization resulted in four stamen types in *C. biscapsularis*, with both fertile and sterile stamens. Tucker^39^ and Marazzi *et al*.^30^ have investigated the androecial development process in *C. biscapsularis* and sibling species. Our RNA-Seq data revealed significant difference in transcriptomic expression among different stamen types. Many more transcripts with very low expression (RPKM ≤ 0.5) were presented in staminodes, while the three fertile stamen sets were overall similar.

In the six comparisons, stamen morphological and structural difference increased from L-M, S-M, L-S, S-St, M-St, to L-St. Screening of differentially expressed genes demonstrated the number of DEGs increased with similar patterns, indicating the molecular signatures underlining morphological differentiation^40^. Transcriptomic analysis identified enriched GO terms relevant to meristem growth regulation, xylem and phloem development in L-S cluster, suggesting tissue growth regulation contributed to the filament length difference. The significant difference of ribosomal-related transcripts between fertile stamens and staminodes demonstrated the lack of protein synthesis in the degenerated stamens, which have lost the ability of pollen producing^7^ (Fig. 1E,F).

### Important gene families were identified relating to intra-floral stamen differentiation

The large number of DEGs between stamen sets implied complex regulatory network controlling stamen size and structure. The bHLH family TFs were highlighted for their role in regulating tapetum-preferential genes^37^, including the AMS-like and DYT1-like TFs. AMS is a key factor for tapetal cell development and post-meiotic microspore formation^36^,^37^. And the *dyt1* mutant of *Arabidopsis* was male sterile with shorter filaments, smaller anthers and no pollen grains^41^, in good agreement with the characteristics of staminodes in *C. biscapsularis*, in which tapetum or microspores were completely absent (Fig. 1E,F). Pathway enrichment analysis revealed the down-regulated *bHLH*-like genes encode the MYC2 proteins in the jasmonate signaling pathway. JAs modulate a number of vital physiological processes, mainly including flower development and fertility, secondary metabolite biosynthesis and defense responses^42^,^43^.

Among the three fertile stamen sets, transcripts encoding auxin response factors (ARFs) were identified as potential genes regulating stamen length. Gibberellin is another essential phytohormone promoting plant cell elongation and division^44^. In our analysis, a gibberellin 20 oxidase 2 (GA 20ox 2)-like gene (Unigene8068) was highlighted. GA 20ox is proved to be the key factor governing the synthesis of bioactive GA1 and GA4^45^.

In angiosperms, MADS-box genes encode a family of transcription factors that are involved in numerous developmental processes, especially in floral identity determination and organ development^17^. Most (19 out of 22) of the identified MADS-box transcripts showed significant down-regulation in the staminodes, in agreement with their roles in floral development and pollen maturation^46^. Interestingly, only one CYC gene (CYC1, Unigene23017) was annotated, with expression highest in staminodes, and lowest in the long stamens. This is well consistent with previous reports in *Antirrhinum* that the asymmetric expression pattern of CYC maintained during flower development, with higher expression in dorsal staminode^47^,^48^. Further functional analyses on screened candidate genes would shed more light on the molecular regulation of stamen development.

In conclusion, the present study established comprehensive relationship between global gene expression and heteranthery in *C. biscapsularis*, representing the largest genetic resource for *Cassia* to date. Complex biological pathways were involved in the morphological differentiation. Molecular components in stamen development and phytohormone signaling were highlighted, which may take essential parts in the intra-floral stamen differentiation. Identified candidate genes regulating tapetum and microspore development would be valuable for male fertile line development in agronomy, especially for fabaceous commercial crops. This is the first study to touch on the genetic basis of heteranthery. In the next step, functional studies on candidate genes would further our understanding in the molecular mechanism of the adaptive evolution in angiosperm flowers. Though our data primarily focus on the middle- to late-stages of stamen development, identified transcripts could have taken pivotal roles in the formation of heteranthery. Despite of the great technical challenges in obtaining primordial stage organs, transcriptomic profiling in flower buds of very early stage in future studies could shed more light on stamen identity determination.

## Methods

### Anatomical analysis of the heteromorphic stamens in *Cassia biscapsularis*

Flower buds at different developmental stages were collected and immediately fixed in 4% glutaraldehyde solution, vacuumed for 2 h, and stored at 4 °C. Samples were then pre-stained with Ehrlichs haematoxylin, and dehydrated through an ethanol series, followed by embedding in paraffin (Leica Microsystems, Germany). Sections of 8 μm in thickness were made by a Leica RM2016 rotary microtome. Sections were examined and photographed using an Olympus BX41 microscope.

### RNA extraction and Illumina sequencing

As we primarily focus on stamen differentiation during organ maturation, flower buds of 5–9 mm in diameter were collected for RNA-Seq. Anatomical and field examinations revealed stamens of this stage were experiencing significant morphological differentiation, including anther size expansion and filament elongation. A total of 240 fresh flower buds were harvested from 20 individuals. The four stamen sets were collected respectively, including the anthers and filaments, frozen in liquid nitrogen immediately, and then stored at −80 °C for RNA extraction (following Onda *et al*.)^49^. Special attention has been paid to avoid RNase contamination.

Total RNA was extracted from the four sets of stamens respectively with the SV Total RNA Isolation System (Promega, USA, Cat.# Z3105). The quantity and quality of RNA were assessed by a NanoDrop 2000 UV-Vis Spectrophotometer (Thermo Scientific, USA).

For RNA-Seq, equivalent quantities of total RNA isolated from different stamen sets were prepared. After the DNase I treatment, magnetic beads with Oligo (dT) were used to isolate mRNA. Mixed with the fragmentation buffer, the mRNA is fragmented into short fragments. Then cDNA is synthesized using the mRNA fragments and random hexamer primers. The suitable fragments are selected for the PCR amplification as templates. During the QC steps, Agilent 2100 Bioanaylzer and ABI StepOnePlus Real-Time PCR System are used in quantification and qualification of the sample library. The libraries were sequenced by Illumina HiSeq™ 2000 at the Beijing Genome Institute (BGI) (Shenzhen, China), with a read length of 100 bp.

Reads with adaptors or unknown nucleotides larger than 5% were removed from the raw reads. And low quality reads (the percentage of low quality bases is more than 20%) have also been discarded.

### *De novo* assembly and functional annotation

Transcriptome *de novo* assembly of each library was carried out with Trinity, a short reads assembling program^35^. Trinity partitions the sequence data into many individual de Bruijn graphs, each representing the transcriptional complexity at a given gene or locus, and then processes each graph independently to extract full-length splicing isoforms and to tease apart transcripts derived from paralogous genes. RNA-seq data were assembled into contigs, scaffolds and finally unigenes by the process.

Annotation analysis provides information of gene expression and functional annotation of All-Unigene in each sample. Unigene sequences are firstly aligned by blastx to protein databases NR (http://www.ncbi.nlm.nih.gov/), Swiss-Prot (http://www.expasy.ch/sprot), KEGG (http://www.genome.ad.jp/kegg/) and COG (http://www.ncbi.nlm.nih.gov/COG/) (e-value < 1.0E-5), and aligned by blastn to nucleotide databases NT (e-value < 1.0E-5), retrieving proteins with the highest sequence similarity with the given unigenes along with their protein functional annotations. For unigenes that cannot be aligned to any of these databases, ESTScan^50^ was used to determine their coding regions and sequence orientation. The Blast2GO program^51^ was used to get GO annotation (http://www.geneontology.org) for unigenes, and metabolic pathways were assigned from species with top BLAST hit in the KEGG database^52^.

### Screening of differentially expressed genes (DEGs)

The RPKM (Reads Per kb per Million reads) method^53^ was used to compare gene expression levels between the transcriptome sequences from different stamen sets. DEGs between any two samples were identified and analyzed on the BGI’s computation platform employing a strict algorithm developed from the significance of digital gene expression profiles^54^. The statistical test results were corrected for multiple testing with the Benjamini–Hochberg false discovery rate (FDR). Sequences were selected as significant DEGs if the FDR ≤ 0.001, and fold change >2 between any two transcriptomes.

### GO classification of DEGs and pathway analysis

All DEGs were mapped to each term of Gene Ontology database, and significantly enriched GO terms were found in DEGs compared to the genome background, taking the corrected-*p*value (FDR) ≤ 0.05 as a threshold. ReviGO was used to visualize the biological processes of enriched GO terms^55^. Enrichment analysis (Fisher’s Exact Test) was performed in Blast2GO to test the significance of enrichment of selected gene families. To identify significantly enriched metabolic pathways or signal transduction pathways with DEGs, KEGG Pathway Analysis were performed with Qvalue ≤ 0.05 as the criteria for pathway selection.

## Additional Information

**How to cite this article**: Luo, Z. *et al*. Transcriptomic analysis of heteromorphic stamens in *Cassia biscapsularis* L. *Sci. Rep.* **6**, 31600; doi: 10.1038/srep31600 (2016).

## Supplementary Material

Supplementary Information

Supplementary Table S1

Supplementary Table S3

## Figures and Tables

**Table 1:** Statistics of sequencing and assembly quality for each library.

		Cb-L	Cb-M	Cb-S	Cb-St
Data production	No. of Clean Reads	74,049,426	89,282,442	74,390,724	84,047,876
	Q20 percentage	97.35%	97.27%	97.34%	97.27%
	N percentage	0.00%	0.00%	0.00%	0.00%
	GC percentage	44.77%	45.27%	45.29%	44.95%
Contigs	Total Number	116,628	118,904	112,425	105,419
	Total Length (nt)	47,175,062	47,630,839	45,260,035	43,269,030
	Mean Length (nt)	404	401	403	410
	Length of N50 (nt)	840	805	776	837
Unigenes	Total Number	78,773	84,297	80,063	78,120
	Total Length (nt)	56,533,899	56,792,745	52,718,950	55,014,933
	Mean Length (nt)	718	674	658	704
	Length of N50 (nt)	1177	1076	1063	1169
