# Supplementary material for: Transcriptomic analysis of heteromorphic stamens in Cassia biscapsularis L
Source: Sci Rep. 2016 Aug 16;6:31600. doi: 10.1038/srep31600 (PMC4985808; doi:10.1038/srep31600)
Supplement: Supplementary Information [file srep31600-s1.pdf]

Supplementary Information

Transcriptomic analysis of heteromorphic stamens in *Cassia biscapsularis* L.

Zhonglai Luo[1], Jin Hu[1,2], Zhongtao Zhao[1] & Dianxiang Zhang [1]*

[1]Key Laboratory of Plant Resources Conservation and Sustainable Utilization, South China Botanical Garden, the Chinese Academy of Sciences, Guangzhou 510650, China.

[2] Shenzhen Park Service, Shenzhen 51800, China

* Correspondence should be addressed to: dx-zhang@scbg.ac.cn

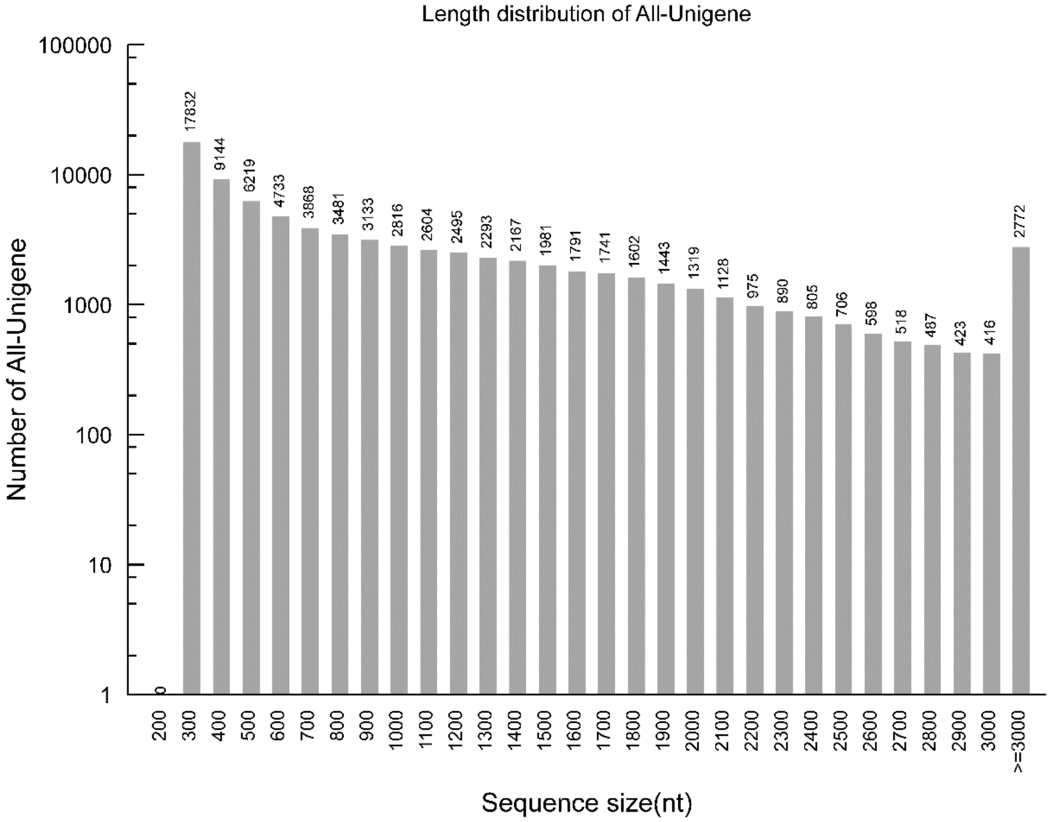

Figure S1. Length distribution of unigenes.

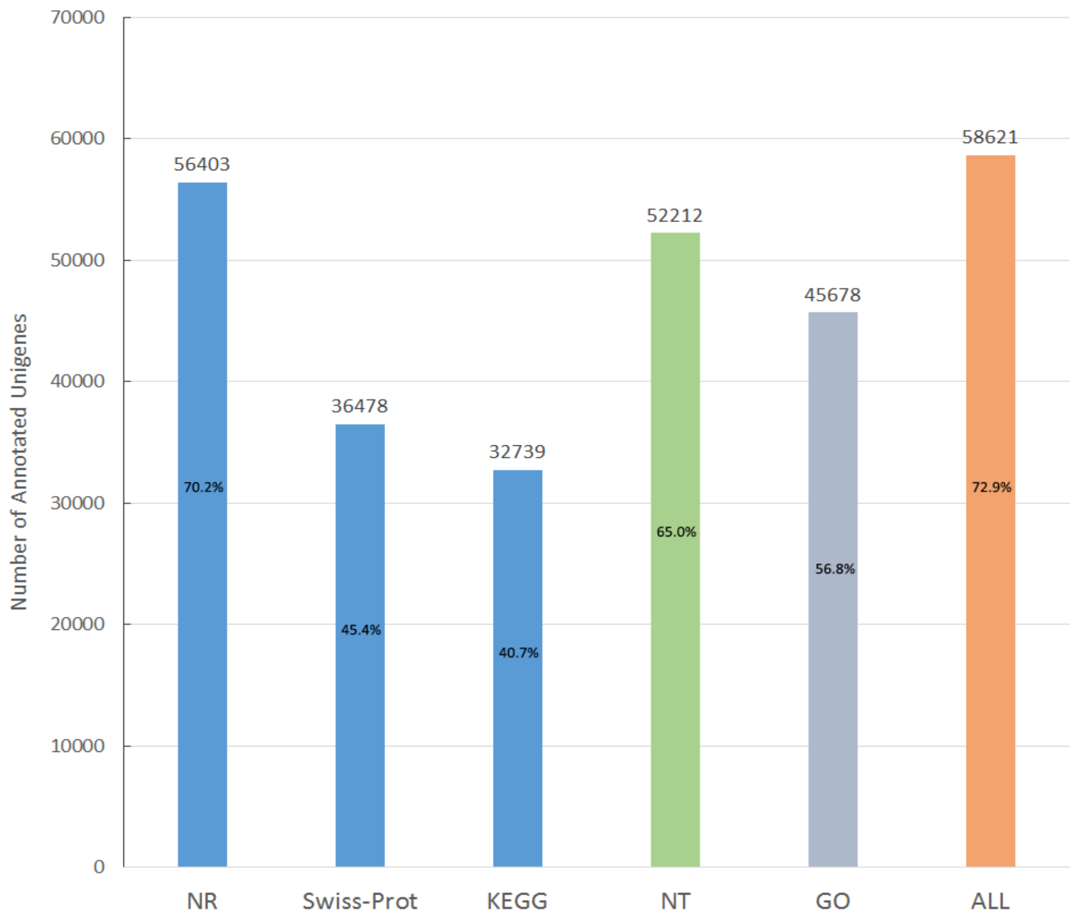

Figure S2. Number of unigenes annotated with different databases.

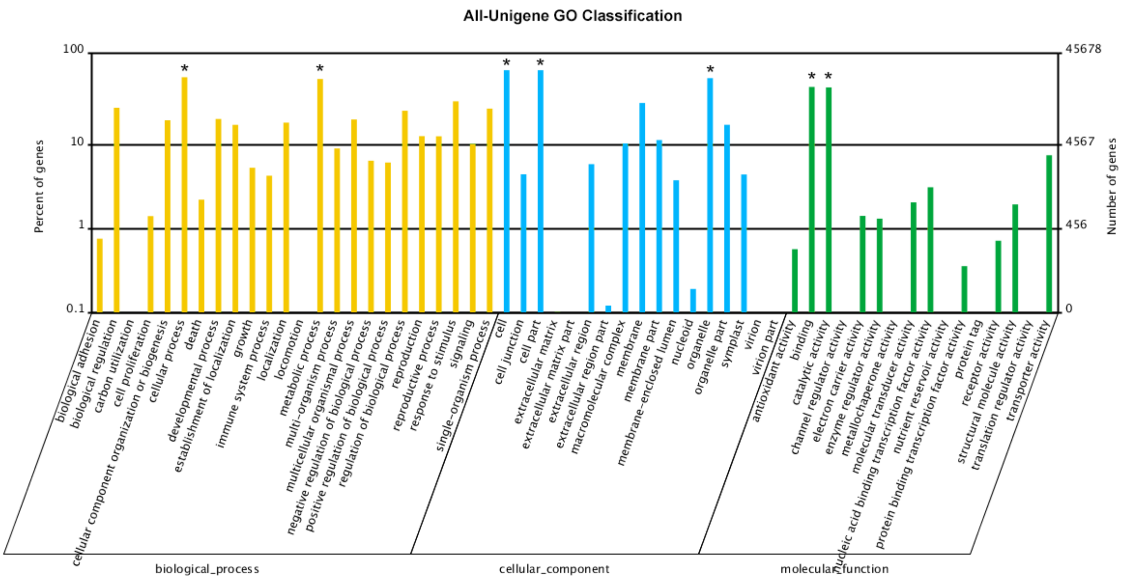

Figure S3. Gene Ontology classifications of assembled non-redundant unigenes. The asterisks indicate the most highly represented GO terms in each ontology.

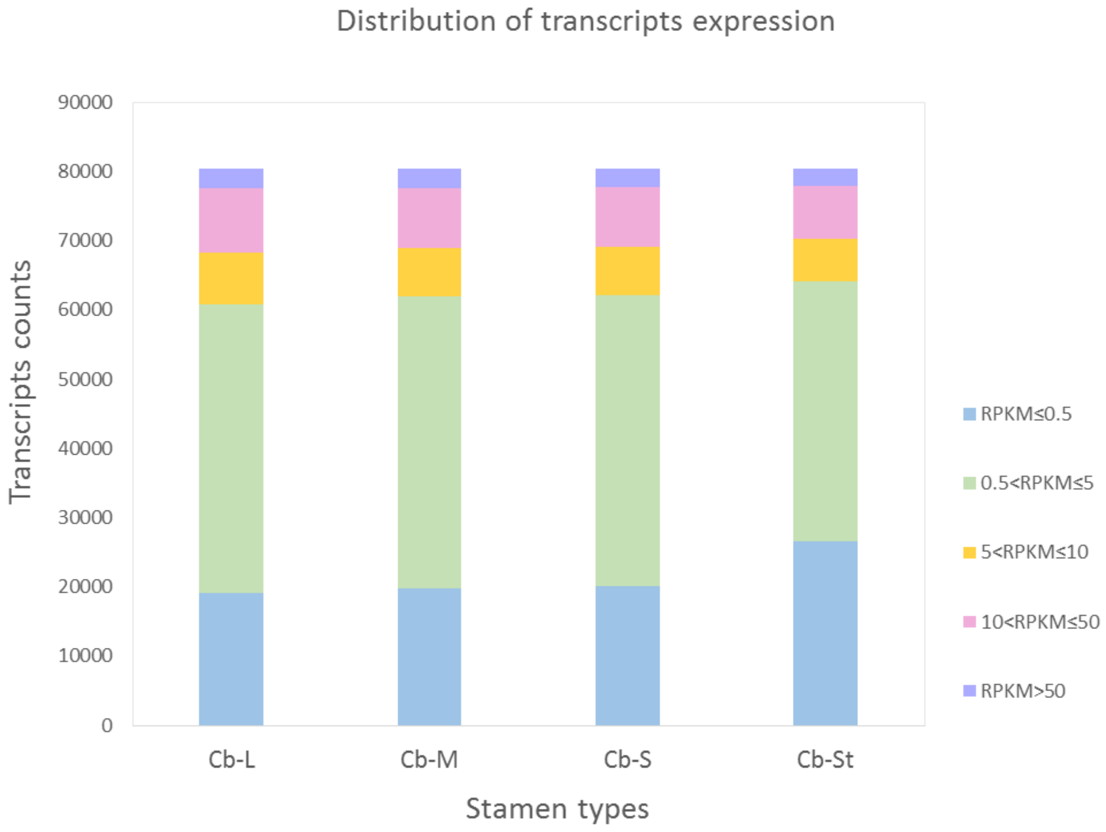

Figure S4. Distribution of RPKM values in different stamen sets.

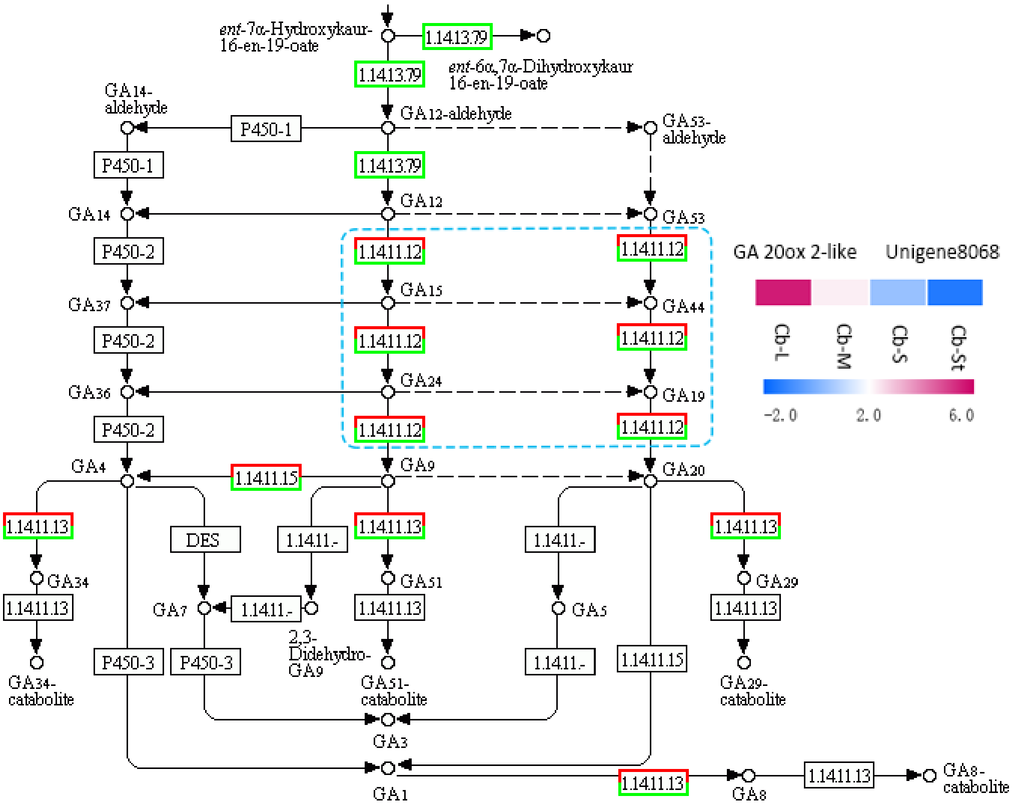

Figure S5. Gibberellin metabolic pathway in *Cassia biscapsularis*. Heat map shows the expression pattern of the key transcript Unigene8068 encoding gibberellin 20 oxidase 2 in different types of stamens (log2-transformed RPKM). Biological processes involving Unigene8068 were highlighted in dashed box.

Supplementary Table S2. Top five significantly enriched GO terms in each comparisons of different stamen sets.

Comparison	GO term	Description	Corrected P-value
Cb-L vs. Cb-M	GO:0005576	extracellular region	5.20e-08
	GO:0048046	apoplast	2.90e-05
	GO:0000418	DNA-directed RNA polymerase IV complex	0.00549
	GO:0009531	secondary cell wall	0.00816
	GO:0000419	DNA-directed RNA polymerase V complex	0.00836
Cb-L vs. Cb-S	GO:0005576	extracellular region	4.00e-13
	GO:0048046	apoplast	1.68e-09
	GO:0009505	plant-type cell wall	1.83e-05
	GO:0043591	external encapsulating structure	7.99e-05
	GO:0005618	cell wall	0.00058
Cb-S vs. Cb-M	GO:0005576	extracellular region	1.05e-15
	GO:0043591	external encapsulating structure	0.00802
	GO:0005618	cell wall	0.01166
		vesicle	0.02744
	GO:0009505	plant-type cell wall	0.03678
Cb-L vs. Cb-St	GO:0044391	ribosomal subunit	6.19e-35
	GO:0022626	cytosolic ribosome	1.93e-31
	GO:0033279	ribosome	2.03e-26
	GO:0044445	cytosolic part	7.58e-25
	GO:0022625	cytosolic large ribosomal subunit	8.05e-23
Cb-M vs. Cb-St	GO:0044391	ribosomal subunit	2.49e-46
	GO:0022626	cytosolic ribosome	4.10e-41
	GO:0033279	ribosome	1.44e-35
	GO:0044445	cytosolic part	1.19e-33
	GO:0022625	cytosolic large ribosomal subunit	1.36e-27
Cb-S vs. Cb-St	GO:0044391	ribosomal subunit	1.21e-40
	GO:0022626	cytosolic ribosome	4.10e-41
	GO:0033279	ribosome	1.44e-35
	GO:0044445	cytosolic part	1.19e-33
	GO:0022625	cytosolic large ribosomal subunit	1.36e-27

Supplementary Table S4. Enrichment test of the bHLH transcription factor family between fertile stamens and staminodes in the DE gene set compared to the total number of TFs (FDR ≤ 0.05 as a threshold).

Comparison	GO term	Description	FDR
Cb-L vs. Cb-St	GO:0001071	nucleic acid binding transcription factor activity	2.69E-03
	GO:0003700	transcription factor activity, sequence-specific DNA binding	2.69E-03
	GO:0048658	anther wall tapetum development	1.21E-04
	GO:0048653	anther development	3.33E-04
	GO:0048443	stamen development	3.33E-04
	GO:0048466	androecium development	3.33E-04
	GO:0048437	floral organ development	8.12E-04
	GO:0048438	floral whorl development	9.71E-04
Cb-M vs. Cb-St	GO:0001071	nucleic acid binding transcription factor activity	1.55E-03
	GO:0003700	transcription factor activity, sequence-specific DNA binding	1.55E-03
	GO:0048658	anther wall tapetum development	8.70E-05
	GO:0048653	anther development	2.40E-04
	GO:0048443	stamen development	2.40E-04
	GO:0048466	androecium development	2.40E-04
	GO:0048437	floral organ development	5.23E-04
Cb-S vs. Cb-St	GO:0001071	nucleic acid binding transcription factor activity	1.56E-03
	GO:0003700	transcription factor activity, sequence-specific DNA binding	1.56E-03
	GO:0048658	anther wall tapetum development	6.84E-05
	GO:0048653	anther development	1.89E-04
	GO:0048443	stamen development	1.89E-04
	GO:0048466	androecium development	1.89E-04
